# Effects of a personalized exercise program on physical function in older patients with rheumatoid arthritis at high risk of sarcopenia: results of a randomized controlled trial

**DOI:** 10.1186/s13075-026-03751-8

**Published:** 2026-02-06

**Authors:** Mie Torii, Akira Onishi, Ryuji Uozumi, Yu Hidaka, Hideo Onizawa, Takayuki Fujii, Koichi Murata, Kosaku Murakami, Masao Tanaka, Yohei Oshima, Hiroki Tanaka, Yuki Urai, Kyosuke Tanigawa, Hiroyuki Yoshitomi, Hideaki Tsuji, Mirei Shirakashi, Ryosuke Hiwa, Ran Nakashima, Kazuko Nin, Ayae Kinoshita, Shuichi Matsuda, Akio Morinobu, Hidenori Arai, Motomu Hashimoto

**Affiliations:** 1 Human Health Sciences, Graduate School of Medicine, Kyoto University, Kyoto, Japan; 2 Graduate School of Nursing, Nara Medical University, Kashihara, Japan; 3 Department of Advanced Medicine for Rheumatic Diseases, Graduate School of Medicine, Kyoto University, Kyoto, Japan; 4 Department of Rheumatology and Clinical Immunology, Graduate School of Medicine, Kyoto University, Kyoto, Japan; 5 Department of Industrial Engineering and Economics, Institute of Science Tokyo, Tokyo, Japan; 6 Department of Biomedical Statistics and Bioinformatics, Kyoto University Graduate School of Medicine, Kyoto, Japan; 7 Department of Immunology Medicine, Shiga General Hospital, Shiga, Japan; 8 Department of Orthopedic Surgery, Graduate School of Medicine, Kyoto University, Kyoto, Japan; 9 Division of Clinical Immunology and Cancer Immunotherapy, Center for Cancer Immunotherapy and Immunobiology, Graduate School of Medicine, Kyoto University, Kyoto, Japan; 10 Rehabilitation Unit, Kyoto University Hospital, Kyoto, Japan; 11 Department of Immunology, Graduate School of Medicine, Kyoto University, Kyoto, Japan; 12 National Center for Geriatrics and Gerontology, Obu, Aichi Japan; 13 Department of Clinical Immunology, Graduate School of Medicine, Osaka Metropolitan University, Osaka, Japan

**Keywords:** Rheumatoid arthritis, Sarcopenia, Randomized controlled trial, Exercise therapy, Short physical performance battery, Short form-12 health survey

## Abstract

**Background:**

Patients with rheumatoid arthritis (RA) are at a higher risk for sarcopenia than the general population. Exercise therapy can improve muscle strength in older adults; however, its efficacy in older patients with RA has not been fully established. This study aimed to evaluate the efficacy of a personalized exercise program on physical function in older patients with RA at high risk for sarcopenia.

**Methods:**

A single-centre, parallel-group, two-arm, superiority randomized controlled trial was conducted in patients with RA aged 60–85 years who were at risk of sarcopenia. The intervention group (*n* = 69) underwent a 16-week personalized exercise program in addition to nutritional guidance and standard care, whereas the control group (*n* = 65) received only nutritional guidance and standard care. The primary outcome was the change in the total Short Physical Performance Battery (SPPB) scores from baseline to week 16.

**Results:**

A total of 140 patients were randomized. Of these, 134 initiated the assigned intervention. There was a 0.2-point difference in SPPB total score from baseline to week 16 between the intervention group (+ 0.4 points) and the control group (+ 0.2 points); 95% confidence interval: -0.1 to 0.5; *p* = 0.206. Regarding the secondary outcomes at week 16, there was a tendency for improvement in the chair-stand test, grip strength, and the mental component score.

**Conclusion:**

The 16-week personalized exercise therapy did not improve the total SPPB scores. However, the intervention may improve standing ability, grip strength, and mental health-related quality of life in older patients with RA at high risk of sarcopenia.

**Trial registration:**

This study was registered with UMINCTR (trial number: UMIN000044930).

**Supplementary Information:**

The online version contains supplementary material available at 10.1186/s13075-026-03751-8.

## Background

Sarcopenia is an age-related syndrome characterized by decreased skeletal muscle mass and loss of muscle strength or physical function. Sarcopenia leads to reduced mobility, increased risk of falls and fractures, decreased activities of daily living (ADL) and quality of life (QoL), substantial healthcare costs, and increased mortality; therefore, it is considered a significant complication [1]. Sarcopenia can be classified into two categories: primary and secondary. Primary sarcopenia is caused by aging, whereas secondary sarcopenia results from inflammatory diseases, malnutrition, or physical inactivity.

Rheumatoid arthritis (RA) is a major cause of secondary sarcopenia. A large-scale epidemiological study using UK Biobank data revealed that RA had the strongest association with sarcopenia among 28 common diseases [2]. In our previous study, we also found that 51% of older patients with RA aged 65 years or older had sarcopenia [3]. This prevalence is markedly higher than the estimated 10% reported in the healthy older population [4]. Patients with RA are especially prone to muscle atrophy due to RA-related inflammatory cytokines, glucocorticoid treatment, and other contributing factors. Additionally, joint pain and deformities reduce muscle mass, muscle strength, and physical activity, exacerbating these conditions owing to reduced movement. Therefore, sarcopenia is a serious complication in patients with RA; however, no effective treatment has been established.

In general, exercise and nutritional therapy are effective in preventing and reducing sarcopenia in older adults [5, 6]. Exercise enhances muscle protein synthesis and exerts anti-inflammatory effects. Moderate-to-high-intensity exercise therapy has been shown to increase muscle strength and mass in healthy older adults [7]. Furthermore, aerobic and high-load exercises have been reported to improve physical function in patients with RA [8]. However, these studies were conducted in predominantly middle-aged patients, and it is not known whether high-intensity training can also improve physical function in older patients with RA who have declining physical function. Furthermore, excessive training may exacerbate disease activity and joint destruction, necessitating careful regulation of exercise intensity [9]. Therefore, exercise therapy for older patients with RA should be carefully tailored to individual patients to avoid adverse events or disease exacerbation. To date, there is no established evidence supporting the efficacy of exercise therapy aimed at reducing sarcopenia in older patients with RA. In this study, we developed a home-based, personalized exercise program that includes resistance and aerobic exercises, with loads adjusted according to the patients’ physical function. Utilizing this program, we conducted a randomized controlled trial (RCT) to evaluate the efficacy of personalized exercise therapy for older patients with RA who were at high risk of sarcopenia.

## Patients and methods

### Study design

This study was a single-centre, parallel-group, healthcare professional/outcome assessor-blinded, superiority RCT conducted at Kyoto University Hospital. The study protocol was approved by the Kyoto University Ethics Committee (approval number: C1545-1) and registered with UMINCTR (trial number: UMIN000044930) [10]. The protocol for this trial was developed according to the Standard Protocol Items: Recommendations for Interventional Trials (SPIRIT) guidelines [11, 12]. The study adhered to the Consolidated Standards of Reporting Trials (CONSORT) guidelines and was conducted in accordance with the principles outlined in the Declaration of Helsinki [13, 14]. Written informed consent was obtained from all patients. The study commenced on January 20, 2022, and ended on July 31, 2023.

### Participants

The inclusion criteria included: (1) age between 60 and 85 years at the time of enrollment, (2) RA diagnosis according to the 2010 American College of Rheumatology/European League Against Rheumatism classification criteria for RA [15], and (3) positive screening test for sarcopenia (grip strength: <28 kg for men and < 18 kg for women, or five times chair-stand test ≥ 12 s) [16]. The exclusion criteria included: (1) inability to walk or stand independently, (2) instruction by a physician to limit exercise, (3) pacemaker use, (4) cognitive impairment (Mini-Mental State Examination score ≤ 23), and (5) any condition deemed contraindicatory for the study by the investigators.

### Randomization and masking

Using a computer-generated random sequence, participants were centrally randomized to either the intervention group or the control group in a 1:1 ratio after screening for eligibility and receiving nutritional guidance. Permuted block randomization stratified by sex and body mass index (BMI; ≥25 or < 25) was used to ensure balance between the groups. Central allocation ensured allocation concealment. Healthcare providers and outcome assessors were blinded to the group assignments; however, it was not possible to blind participants due to the nature of the exercise intervention. To assess the maintenance of blinding among healthcare providers, a questionnaire was administered after the completion of the 16-week exercise program.

### Intervention

The intervention group received individualized exercise programs and nutritional guidance in addition to usual care, whereas the control group received only nutritional guidance in addition to standard care [10]. In this study, standard care referred to regular assessment of disease activity, adjustment of pharmacological therapy, and provision of general lifestyle or self-management guidance by the treating physician. The exercise program, designed for home-based performance, included resistance exercises and aerobic exercises with load management. For resistance exercise, the program consisted of six types for the lower limbs and two types for the fingers. Resistance exercises were scheduled three times a week on non-consecutive days, for 30–60 min per session. Exercise intensity was set at 50–70% of maximal strength, targeting a perceived exertion of 5–6 (“hard”) on the Borg scale [17]. In accordance with the study protocol, research nurses who had been trained by physical therapists instructed participants on how to perform each exercise. Participants also received written instructional materials, including photographs and key instructions for correct exercise performance, precautions, and prescribed repetitions. Exercise performance and intensity adjustments were reviewed every 4 weeks during monthly clinic visits. Adherence to resistance exercises was assessed during monthly face-to-face visits, at which trained exercise instructors (research nurses trained by physical therapists) reviewed the exercise log sheets, verified the frequency of performance, directly observed exercise performance and form, and adjusted training load as needed to address under- or over-loading. With regard to aerobic exercise, individualized step-count goals were established according to the study protocol, aiming to increase baseline step counts by 20% during the first 8 weeks and by an additional 20% during the subsequent 8 weeks. The participants were instructed to record their exercise activities and step counts throughout the intervention. To maintain adherence across both resistance and aerobic components, trained exercise instructors conducted face-to-face meetings every 4 weeks to review implementation status and provide individualized feedback, while outcome assessors remained blinded to group allocation. Exercise therapy protocols for this study were developed and monitored by experienced physical therapists (Y.O. and H.Y.), who created personalized programs based on previous exercise programs [18–20]. The details are described in our protocol paper [10].

Nutritional guidance included: (1) an overview of nutrients, such as proteins, that are beneficial for preventing sarcopenia; (2) information on the protein content of various foods; (3) guidelines for appropriate protein intake; and (4) easy-to-prepare recipes. This guidance was specifically developed by dieticians for the study and provided to research nurses under the guidance of the dieticians.

### Compliance with the exercise program

Participants were instructed to record their exercise activities and Borg Scale ratings on log sheets, which were reviewed every 4 weeks and collected at the end of the study. Additionally, activity monitors were worn every 8 weeks to verify adherence to the exercise program.

### Outcomes

Outcome assessments were conducted at baseline (0 week) and during follow-up visits (8 and 16 weeks). The primary outcome was the change in Short Physical Performance Battery (SPPB) scores from baseline to 16 weeks [21, 22]. The SPPB is a physical function assessment in the diagnostic criteria for sarcopenia [16]. It evaluates physical function using balance, gait speed, and chair-stand tests, with scores ranging from 0 to 12, where higher scores indicate better physical function. Secondary outcomes included the following explanatory variables: (1) each component of the SPPB (balance, gait speed, and chair-stand tests); (2) skeletal muscle index; (3) grip strength; (4) physical activity evaluated with the International Physical Activity Questionnaire (IPAQ) [23, 24]; (5) RA disease activity assessed with the Disease Activity Score 28 joints (DAS28-CRP) [25], Clinical Disease Activity Index (CDAI) [26], and Simplified Disease Activity Index (SDAI) [27]; (6) physical disability assessed using the Health Assessment Questionnaire (HAQ) [28]; (7) overall health-related QoL evaluated by the EuroQoL-5 Dimension-3 Level (EQ-5D-3 L) [29] and 12-Item Short-Form Health Survey (SF-12) [30, 31]; and (8) subjective symptoms (depression and anxiety) evaluated with the Hospital Anxiety and Depression Scale (HADS) [32, 33].

### Safety evaluation

Adverse events were monitored monthly, including severe joint pain, severe headaches, falls, fractures, and the onset or worsening of physical or mental symptoms. All adverse events were recorded in case report forms (CRFs).

### Statistical analysis

Based on the results of previous studies [34–36], we estimated that 128 patients would yield at least 80% power to detect an effect size of 0.5 points in SPPB scores at 16 weeks between the intervention and control groups (primary outcome) at a two-sided significance level of 0.05. To account for an anticipated attrition rate of 20%, we planned to enrol 160 patients. However, we terminated the recruitment at 140 patients because the attrition rate was lower than expected.

All analyses were performed using the intention-to-treat principle. No interim analyses were planned. A linear mixed-effects model for repeated measures was used to estimate the least-squares mean (LSM) difference in SPPB score changes from baseline to 8 and 16 weeks. This model assumed that the primary outcome data were missing at random and included the study group, time points, baseline scores, age at baseline, and the interaction between the study group and time points. Missing data were not imputed. The primary time point was set at 16 weeks. No adjustments were made for multiplicity. Mixed-effects models for repeated measures were considered for secondary outcomes. A subgroup analysis was conducted using the DAS28-CRP category (< 2.6 or ≥ 2.6). All analyses were performed using SAS software version 9.4 (SAS Institute, Cary, NC).

## Results

### Participants

**Fig. 1:**
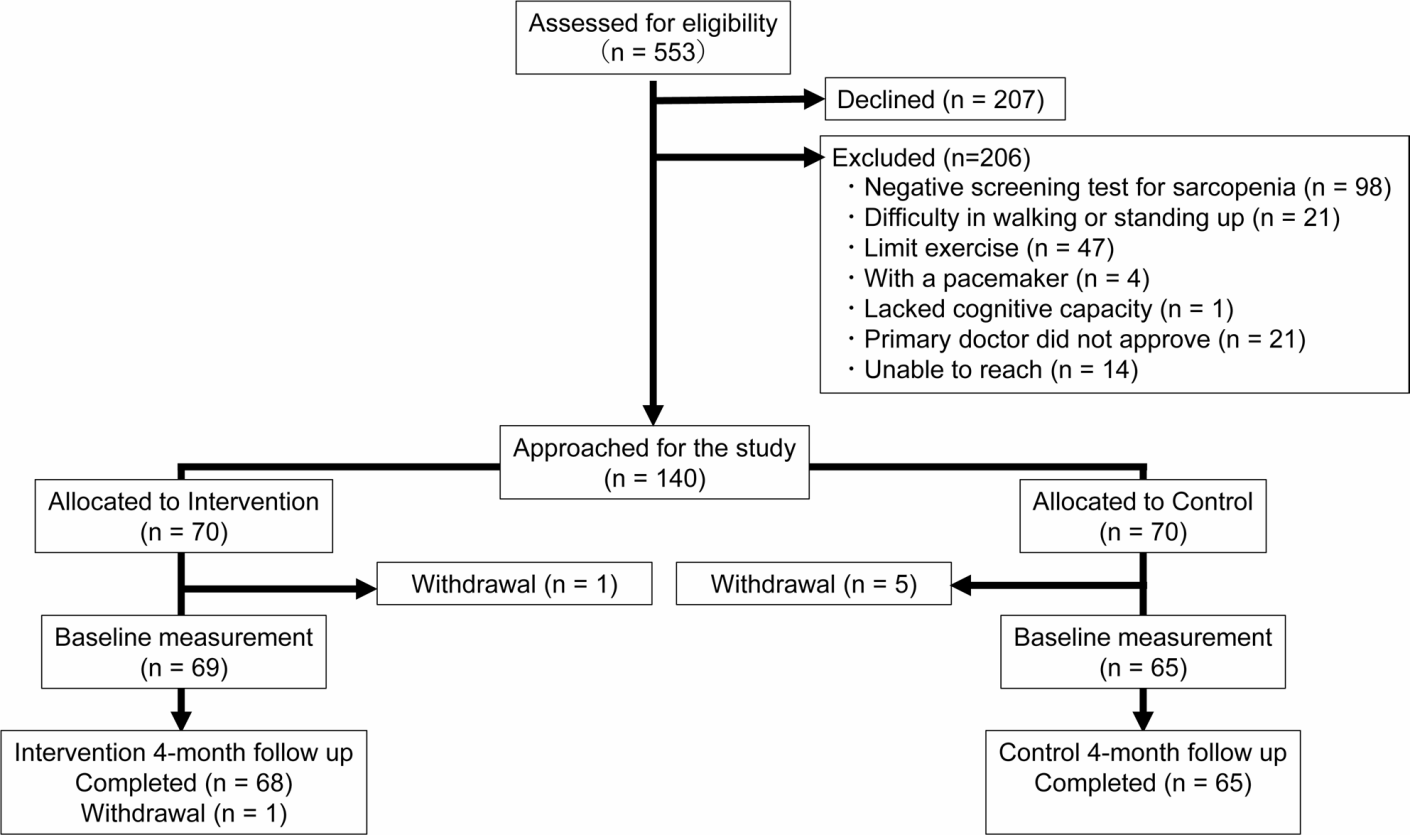
Study flow diagram. A total of 134 participants were included in the intention-to-treat analysis. Intervention: exercise program and nutritional guidance in addition to standard care; Control: nutritional guidance in addition to standard care

A total of 553 patients with RA were screened, and 140 were randomly assigned to the intervention (70 patients) and control (70 patients) groups (Fig. 1). Among the randomized patients, one patient in the intervention group and five in the control group withdrew consent before baseline measurements, leaving 134 participants (69 in the intervention group and 65 in the control group) for the intention-to-treat analysis. During the 4-week post-intervention period, one patient in the intervention group dropped out due to personal reasons. The baseline characteristics of the patients included in the intention-to-treat population are shown in Table 1.

**Table 1:** Baseline participant demographics and disease characteristics

	All participants *n* = 134	Intervention *n* = 69	Control *n* = 65
Age (years), median (IQR)	73.5 (68.0-81.5)	72.0 (67.0–78.0)	74.0 (69.0–76.0)
Sex, female %, (*n*)	79.1 (106)	79.7 (55)	78.4 (51)
Duration of RA (years), median (IQR)	17.5 (10.0-29.3)	15.0 (9.5–28.5)	19.0 (10.0-30.5)
DAS28-CRP (score), median (IQR)	1.8 (1.5–2.7)	1.8 (1.5–2.6)	1.9 (1.5–2.7)
CDAI (score), median (IQR)	4.4 (1.9–7.6)	4.7 (1.5–4.7)	3.8 (2.3-8.0)
SDAI (score), median (IQR)	4.6 (2.1–7.8)	4.8 (1.6–7.5)	4.2 (2.5–8.9)
HAQ-DI, median (IQR)	0.6 (0.3–1.1)	0.6 (0.3–1.1)	0.6 (0.1–1.1)
Methotrexate use, %, (*n*)	57.5 (77)	55.1 (38)	60.0 (19)
Glucocorticoids use, %, (*n*)	22.4 (30)	23.2 (16)	21.5 (14)
Biological and DMARDs use, %, (*n*)	61.9 (83)	68.1 (47)	55.4 (36)
BMI (score), mean ± SD	21.7 ± 3.6	22.0 ± 3.8	21.4 ± 3.4
SMI (kg/m^2^), mean ± SD	5.8 ± 0.8	5.9 ± 0.9	5.8 ± 0.8
Right hand grip (kg), mean ± SD	15.2 ± 6.8	15.0 ± 7.2	15.5 ± 6.5
Left hand grip (kg), mean ± SD	15.0 ± 6.6	14.7 ± 6.6	15.4 ± 6.5
SPPB total score, mean ± SD	10.7 ± 1.7	10.6 ± 1.8	10.8 ± 1.7
SPPB balance score, mean ± SD	3.6 ± 0.7	3.6 ± 0.7	3.6 ± 0.6
SPPB gait speed score, mean ± SD	3.7 ± 0.6	3.7 ± 0.6	3.7 ± 0.6
SPPB chair-stand test score, mean ± SD	3.4 ± 0.9	3.3 ± 0.9	3.5 ± 0.9

*DAS28* disease activity score using 28 joints, *CDAI* Clinical Disease Activity Index, *SDAI* Simplified Disease Activity Index, *HAQ-DI* Health Assessment Questionnaire Disability Index, *DMARDs* disease-modifying anti-rheumatic drugs, *BMI* body mass index, *SMI* skeletal muscle mass index, *SPPB* Short Physical Performance Battery, *SD* standard deviation, *IQR* interquartile range

At baseline, the prevalence of sarcopenia was 50.7% (35/69) in the intervention group and 52.3% (34/65) in the control group.

### Primary outcome

**Fig. 2:**
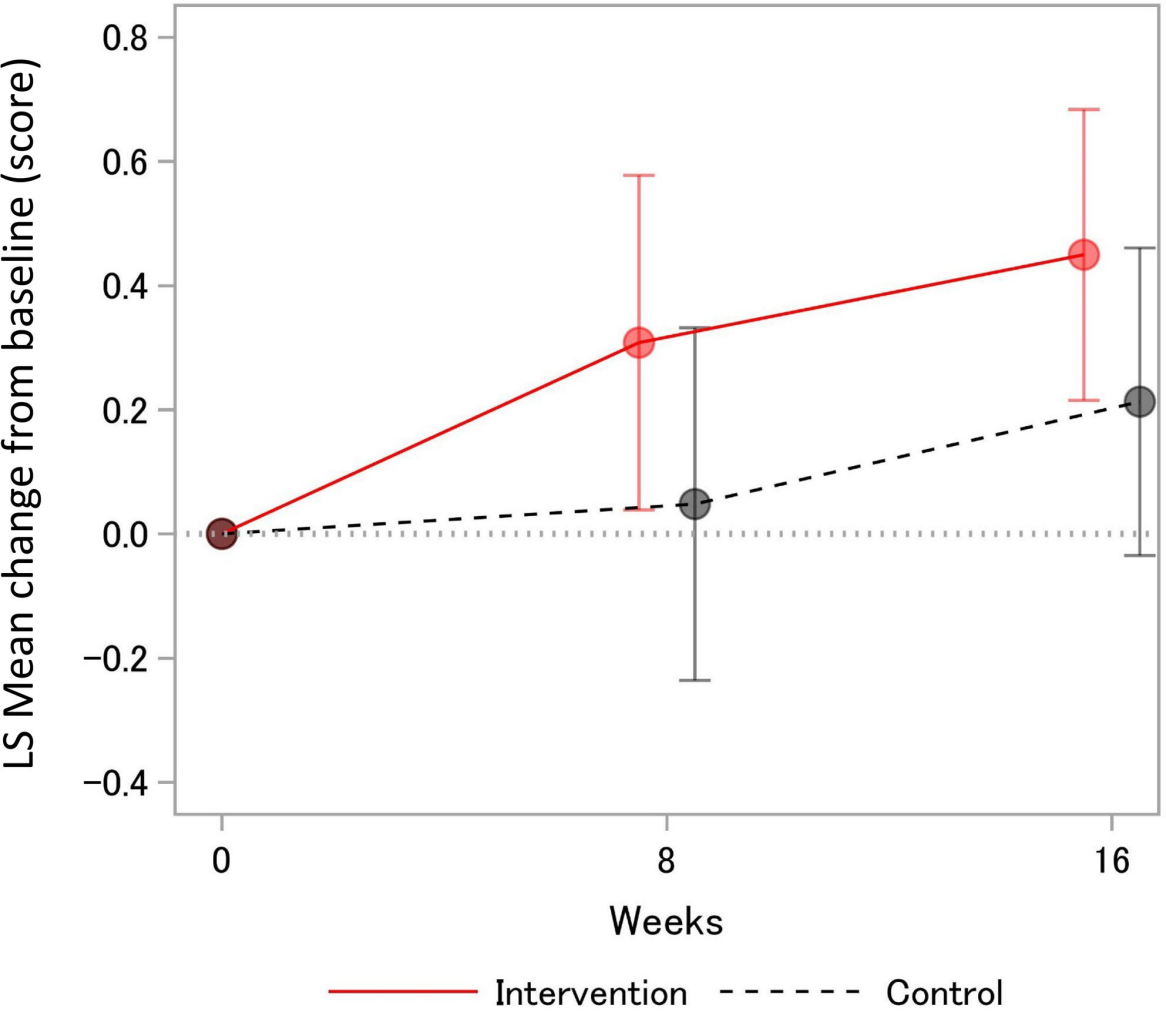
LSM Change in SPPB total scores from baseline. LSM, least-squares mean; SPPB, Short Physical Performance Battery

Figure 2; Table 2 show the changes in LSM from baseline to each evaluation point. The changes in the total SPPB score from baseline to week 16 were + 0.4 points in the intervention group and + 0.2 points in the control group. The difference in SPPB score change between the two groups was 0.2 points (95% confidence interval [CI]: -0.1 to 0.5; *p* = 0.206), which did not reach significance. The difference was similar at 8 weeks (point estimate: 0.2; 95% CI: -0.2 to 0.5). In the subgroup analysis of patients with remission (DAS28-CRP < 2.6), the LSM difference in the total SPPB score from baseline to 16 weeks was 0.3 points (95% CI: 0.0 to 0.6) (Additional File 1).

**Table 2:** LSM changes in SPPB, SMI, and hand grip strength at 8 and 16 weeks

Variables	Week	Intervention (*n* = 69)			Control (*n* = 65)			Comparison between groups	
		*N*	Mean (SD)	LSM changes (95% CI)	*N*	Mean (SD)	LSM changes (95% CI)	Difference in LSM changes (95% CI)	*p*-value
SPPB	0	69	10.7 (1.7)		63	10.8 (1.7)			
total score	8	66	11.0 (1.6)	0.2 (0.0, 0.5)	61	10.9 (1.5)	0.1 (-0.2, 0.3)	0.2 (-0.2, 0.5)	0.358
	16	68	11.1 (1.7)	0.4 (0.2, 0.6)	62	11.0 (1.3)	0.2 (0.0, 0.4)	0.2 (-0.1, 0.5)	0.206
SPPB	0	69	3.6 (0.7)		63	3.6 (0.6)			
balance score	8	67	3.6 (0.7)	0.0 (-0.1, 0.2)	62	3.6 (0.6)	0.0 (-0.2, 0.1)	0.0 (-0.2, 0.2)	0.868
	16	68	3.6 (0.8)	0.0 (-0.1, 0.2)	62	3.7 (0.6)	0.0 (-0.1, 0.2)	0.0 (-0.2, 0.2)	0.841
SPPB	0	69	3.7 (0.6)		65	3.8 (0.5)			
gait speed score	8	67	3.8 (0.5)	0.1 (0.0, 0.1)	63	3.9 (0.4)	0.1 (0.1, 0.2)	0.0 (-0.1, 0.1)	0.562
	16	68	3.8 (0.5)	0.1 (0.0, 0.2)	64	3.9 (0.4)	0.1 (0.1, 0.2)	0.0 (-0.1, 0.1)	0.944
SPPB	0	69	3.3 (1.0)		65	3.5 (0.9)			
chair-stand test	8	67	3.5 (1.0)	0.2 (0.0, 0.3)	63	3.4 (1.1)	0.0 (-0.2, 0.2)	0.2 (-0.1, 0.4)	0.236
score	16	68	3.6 (0.8)	0.3 (0.1, 0.4)	64	3.5 (0.9)	0.1 (-0.1, 0.2)	0.2 (0.0, 0.4)	0.022
Right hand grip	0	69	15.0 (7.2)		65	15.5 (6.5)			
	8	67	15.5 (7.1)	0.5 (-0.1, 1.1)	64	15.1 (6.5)	-0.3 (-1.0, 0.3)	0.8 (0.0, 1.7)	0.062
	16	67	15.4 (7.7)	0.3 (-0.3, 0.9)	65	14.8 (6.4)	-0.7 (-1.3, -0.1)	1.0 (0.1, 1.8)	0.025
Left hand grip	0	69	14.7 (6.6)		65	15.4 (6.5)			
	8	68	14.9 (7.0)	0.3 (-0.2, 0.8)	63	14.7 (6.5)	-0.5 (-1.0, 0.0)	0.8 (0.0, 1.5)	0.039
	16	67	14.6 (7.2)	-0.1 (-0.7, 0.6)	65	14.7 (7.0)	-0.7 (-1.3, 0.0)	0.6 (-0.3, 1.5)	0.180
SMI	0	69	5.9 (0.9)		65	5.8 (0.8)			
	8	67	5.9 (0.9)	0.0 (-0.1, 0.0)	64	5.8 (0.9)	0.0 (-0.1,0.0)	0.0 (-0.1, 0.1)	0.673
	16	68	5.8 (0.8)	0.0 (-0.1, 0.0)	65	5.7 (0.9)	-0.1 (-0.1, 0.0)	0.0 (0.0, 0.1)	0.289

*SPPB* Short Physical Performance Battery, *SMI* skeletal muscle mass index, *LSM* least-squares mean, *CI* confidence interval

### Secondary outcomes

Tables 2, 3 and 4 show the changes in the LSM of secondary outcomes from baseline to the evaluation time points. The differences in chair-stand test score change from baseline between the two groups were 0.2 points (95% CI: -0.1 to 0.4) at 8 weeks and 0.2 points (95% CI: 0.0 to 0.4) at 16 weeks. The group differences in right-hand grip score change from baseline were + 0.8 points (95% CI: 0.0 to 1.7) at 8 weeks and + 1.0 points (95% CI: 0.1 to 1.8) at 16 weeks (Table 2). In addition to the ordinal SPPB scores, raw performance measures for SPPB balance tests (semi-tandem stand and tandem stand, seconds), gait speed (m/s), and chair stand time (seconds) were examined to assess item-level changes. The changes observed in these raw measures were directionally consistent with those obtained using the SPPB scores (Additional File 2). Among overall health-related QoL, a trend toward improvement was observed in the SF-12, specifically in the vitality (VT_N), mental health (MH_N), and 3MCS (mental health) components. The group differences in the VT-N score change from baseline were + 1.3 points (95% CI: -1.4 to 3.9) at 8 weeks and + 3.5 points (95% CI: 0.8 to 6.1) at 16 weeks. The group differences in the MH_N score from baseline were + 1.0 points (95% CI: -1.5 to 3.6) at 8 weeks and + 2.8 points (95% CI: 0.2 to 5.5) at 16 weeks. The group differences in the 3MCS score from baseline were + 1.6 points (95% CI: -0.7 to 3.9) at 8 weeks and + 3.4 points (95% CI: 0.9 to 5.9) at 16 weeks (Table 3). Additionally, the group difference in the HADS-depression score from baseline was − 0.5 points (95% CI: -1.4 to 0.3) at 8 weeks and − 1.1 points (95% CI: -2.0 to -0.1) at 16 weeks (Table 4). No worsening of disease activity was observed in the intervention group (Additional File 3).

**Table 3:** LSM changes from baseline in overall health-related QoL at 8 and 16 weeks

Variables	Week	Intervention (*n* = 69)			Control (*n* = 65)			Comparison between groups	
		*N*	Mean (SD)	LSM changes (95% CI)	*N*	Mean (SD)	LSM changes (95% CI)	Difference in LSM changes (95% CI)	*p*-value
EQ-5D	0	68	0.8 (0.2)		64	0.8 (0.2)			
	8	68	0.8 (0.2)	0.0 (0.0, 0.0)	64	0.8 (0.2)	0.0 (0.0, 0.0)	0.0 (0.0, 0.1)	0.428
	16	68	0.8 (0.2)	0.0 (0.0, 0.0)	65	0.8 (0.2)	0.0 (0.0, 0.0)	0.0 (-0.1, 0.1)	0.834
PF_N	0	67	42.5 (11.6)		65	42.8 (11.0)			
	8	68	42.1 (13.7)	-0.3 (-2.3, 1.8)	64	43.6 (10.8)	0.9 (-1.2, 2.9)	-1.1 (-4.0, 1.8)	0.448
	16	68	43.2 (12.7)	0.7 (-1.5, 3.0)	65	42.8 (11.7)	0.1 (-2.2, 2.4)	0.6 (-2.6, 3.8)	0.698
RP_N	0	67	44.8 (9.4)		65	41.7 (10.8)			
	8	68	43.9 (10.3)	-0.2 (-2.5, 2.1)	64	42.2 (10.3)	-0.2 (-2.5, 2.1)	0.0 (-3.2, 3.3)	0.983
	16	68	43.0 (10.5)	-1.0 (-3.3, 1.2)	65	43.1 (10.7)	0.8 (-1.5, 3.1)	-1.8 (-5.0, 1.4)	0.274
BP_N	0	67	43.3 (10.4)		65	42.7 (9.9)			
	8	68	43.2 (11.2)	0.2 (-2.1, 2.4)	64	42.8 (11.1)	-0.1 (-2.4, 2.2)	0.3 (-2.9, 3.5)	0.855
	16	68	43.8 (10.9)	1.2 (-0.8, 3.2)	65	45.1 (10.4)	2.3 (0.3, 4.4)	-1.1 (-4.0, 1.7)	0.430
GH_N	0	67	47.4 (9.4)		65	46.1 (8.9)			
	8	68	47.8 (9.6)	1.0 (-0.8, 2.9)	64	46.8 (8.4)	0.3 (-1.6, 2.1)	0.8 (-1.9, 3.4)	0.564
	16	68	47.8 (9.4)	1.0 (-1.0, 2.9)	65	47.4 (9.0)	1.0 (-1.0, 2.9)	0.0 (-2.8, 2.8)	0.995
VT_N	0	67	51.1 (10.0)		64	52.1 (9.4)			
	8	67	51.3 (9.7)	0.0 (-1.8, 1.9)	64	50.6 (8.6)	-1.3 (-3.2, 0.6)	1.3 (-1.4, 3.9)	0.335
	16	68	53.6 (9.5)	2.4 (0.6, 4.3)	65	50.6 (9.0)	-1.0 (-2.9, 0.8)	3.5 (0.8, 6.1)	0.010
SF_N	0	67	51.1 (7.3)		65	50.1 (8.6)			
	8	67	51.4 (7.9)	0.6 (-1.5, 2.6)	64	49.7 (9.6)	-0.8 (-2.8, 1.2)	1.4 (-1.5, 4.2)	0.345
	16	68	51.3 (7.7)	0.7 (-1.4, 2.8)	65	48.2 (10.9)	-2.2 (-4.3, 0.0)	2.9 (-0.2, 5.9)	0.063
RE_N	0	67	49.2 (8.9)		65	46.5 (10.4)			
	8	68	48.2 (10.2)	-0.3 (-2.5, 1.9)	64	45.9 (9.4)	-1.4 (-3.6, 0.9)	1.1 (-2.1, 4.2)	0.504
	16	68	48.2 (9.4)	-0.1 (-2.4, 2.1)	65	45.9 (10.8)	-1.3 (-3.5, 1.0)	1.1 (-2.0, 4.3)	0.477
MH_N	0	67	54.5 (8.0)		65	54.2 (8.6)			
	8	67	55.2 (9.8)	0.7 (-1.1, 2.5)	64	54.0 (8.8)	-0.4 (-2.2, 1.4)	1.0 (-1.5, 3.6)	0.420
	16	68	56.2 (8.7)	1.6 (-0.3, 3.5)	65	53.0 (8.9)	-1.2 (-3.1, 0.7)	2.8 (0.2, 5.5)	0.037
3PCS	0	67	38.5 (11.7)		64	38.1 (11.3)			
	8	67	38.1 (13.0)	-0.2 (-2.4, 1.9)	64	39.4 (10.4)	1.2 (-1.0, 3.4)	-1.4 (-4.5, 1.7)	0.366
	16	68	38.2 (12.5)	0.1 (-2.3, 2.4)	65	40.8 (11.9)	2.8 (0.4, 5.2)	-2.7 (-6.1, 0.6)	0.112
3MCS	0	67	53.8 (7.8)		64	54.8 (7.5)			
	8	67	54.7 (7.6)	0.9 (-0.7, 2.5)	64	54.0 (8.3)	-0.7 (-2.4, 0.9)	1.6 (-0.7, 3.9)	0.167
	16	68	56.4 (8.0)	2.6 (0.9, 4.4)	65	53.9 (8.1)	-0.8 (-2.5, 1.0)	3.4 (0.9, 5.9)	0.008
3RCS	0	67	51.8 (8.5)		64	48.9 (10.2)			
	8	67	51.0 (9.9)	-0.2 (-2.4, 2.0)	64	48.3 (9.2)	-1.3 (-3.6, 0.9)	1.1 (-2.0, 4.3)	0.477
	16	68	50.1 (8.8)	-1.0 (-3.1, 1.2)	65	47.2 (10.6)	-2.5 (-4.7, -0.4)	1.6 (-1.5, 4.6)	0.313

*EQ-5D-3 L* Euro- QoL-5 Dimension-3 Level, *SF-12* 12-Item Short-Form Health Survey, *LSM* least-squares mean, *PF_N* Physical Functioning - Norm-based score, *RP_N* Role Physical-Norm-based score, *BP_N* Bodily Pain-Norm-based score, *GH_N* General Health- Norm-based score, *VT_N* Vitality Norm-based score, *SF_N* Social Functioning-Norm-based score, *RE_N* Role Emotional- Norm-based score, *MH_N* Mental Health- Norm-based score, *3PCS* 3 Physical Component Summary, *3MCS* Mental Component Summary, *3RCS* Role Component Summary, *CI* confidence interval

**Table 4:** LSM changes from baseline in HADS score at 8 and 16 weeks

Variables	Week	Intervention (*n* = 69)			Control (*n* = 65)			Comparison between groups	
		*N*	Mean (SD)	LSM changes (95% CI)	*N*	Mean (SD)	LSM changes (95% CI)	Difference is LSM changes (95% CI)	*p*-value
HADS-A	0	68	4.4 (3.3)		65	4.0 (3.0)			
	8	68	4.3 (3.9)	-0.2 (-0.7, 0.4)	64	3.5 (3.0)	-0.4 (-0.9, 0.2)	0.2 (-0.6, 1.0)	0.605
	16	67	3.8 (3.6)	-0.5 (-1.1, 0.0)	65	3.9 (3.5)	-0.1 (-0.7, 0.5)	-0.5 (-1.3, 0.4)	0.278
HADS-D	0	69	5.1 (3.5)		65	5.6 (3.9)			
	8	68	5.4 (3.4)	0.1 (-0.5, 0.7)	64	6.1 (3.9)	0.7 (0.1, 1.3)	-0.5 (-1.4, 0.3)	0.209
	16	68	4.8 (3.6)	-0.5 (-1.1, 0.2)	65	6.2 (4.2)	0.6 (-0.1, 1.3)	-1.1 (-2.0, -0.1)	0.026

*HADS-A* Hospital Anxiety and Depression Scale-Anxiety, *HADS-D* Hospital Anxiety and Depression Scale-Depression, *LSM* least-squares mean, *CI* confidence interval

### Adverse events

No serious adverse events associated with the exercise therapy were observed. A total of 39 adverse events were reported during the follow-up period (22 in the intervention group and 17 in the control group). These events included falls (20 in the intervention group and 15 in the control group) and fractures (two in each group). Notably, these adverse events occurred independently of the exercise program sessions.

## Discussion

To the best of our knowledge, this RCT is the first to evaluate improvements in physical function following a personalized exercise program in older patients with RA at high risk for sarcopenia. After 16 weeks of exercise, there were notable trends toward improvement in the chair-stand test, grip strength, and mental health-related QoL. Furthermore, the exercise intervention did not exacerbate RA disease activity, and no adverse effects related to the exercise therapy were reported. The intervention effect did not differ between patients with and without remission, suggesting that responsiveness to the exercise program may not be strongly influenced by disease activity status; however, this finding should be interpreted with caution, given the exploratory nature of the subgroup analysis.

This study did not achieve statistical significance in the primary outcome of physical function observed at 16 weeks of exercise intervention. There are five possible reasons for this finding. First, the duration of the exercise intervention may have been insufficient for older patients with RA to experience significant improvements in physical function. A chronic inflammatory state or aging may inhibit muscle protein synthesis, leading to delayed physical function recovery compared with healthy individuals [37–39]. Consequently, a longer intervention period may be required to observe measurable improvements in physical performance, given that previous 16-week exercise interventions in patients with RA have demonstrated improvements in muscle strength, pain, and self-reported functional outcomes, while improvements in objective physical performance measures have been variable across studies [40, 41]. Longer-term exercise interventions have also been evaluated in patients with RA; for example, an RCT demonstrated that 24 weeks of high-intensity progressive resistance training improved lean body mass and objective physical function without exacerbating disease activity [42]. Second, this study screened patients at high risk of sarcopenia according to the Asian Working Group for Sarcopenia 2019 criteria, using grip strength and stand-up tests. We used the change in SPPB score as the primary outcome because the SPPB is an internationally validated and widely recommended measure for assessing physical function in patients with sarcopenia and frailty, and has been widely used in populations with chronic inflammatory diseases [43, 44]. Although the SPPB has been reported to be susceptible to the ceiling effect in high-functioning populations, the present study specifically targeted older patients with RA who were at high risk of sarcopenia and functional vulnerability rather than high physical performance. Therefore, a pronounced ceiling effect was not anticipated in this study population. Nevertheless, approximately half of the participants classified as high risk for sarcopenia achieved the maximum SPPB score at baseline, which may have partially limited the magnitude of observable improvement in physical function during the intervention period. Third, adherence to the exercise program may have affected the results. In a post-hoc exploratory analysis, the mean overall adherence rate in our study was 89.4% for lower-limb exercises and 86.4% for hand exercises. Moreover, patients in the intervention group with an adherence rate of more than 80% showed improvements in their total SPPB score at 8 weeks (LSM change from baseline: 0.2 points; 95% CI: 0.0 to 0.5) and 16 weeks (LSM change from baseline: 0.5 points; 95% CI: 0.1 to 0.9). This suggests that the intervention may be effective in improving physical function in older patients with RA, and that higher adherence may be associated with favourable outcomes. Fourth, we anticipated an effect size of 0.5 following previous studies; however, the effect size in our study was smaller than expected. Thus, the smaller effect size may have been assumed to calculate a larger sample size. Fifth, exercise intensity in this trial was adjusted using the subjective Borg Rating of Perceived Exertion scale, without incorporating objective indicators such as heart rate or oxygen consumption. Although this approach enhances feasibility in routine clinical practice, the use of objective indicators may have allowed for a more precise and individualized adjustment of exercise intensity. In this study, we found a tendency for improvement in mental health-related QoL assessed by the SF-12 and in depression assessed by the HADS, compared with baseline and the control group. Similar improvements in mental health-related QoL through exercise have been reported in patients with hypothyroidism and in frail institutionalized older adults [45–47]. Additionally, studies have shown that aerobic and resistance exercise interventions improve mental health and vitality, as evaluated using the SF-36 in individuals with type 2 diabetes [48]. These results indicate that exercise therapy may play a valuable role in supporting emotional well-being and vitality.

This study has several strengths. First, strict blinding was achieved during the outcome evaluations to minimize information bias. This trial was designed with a rigorous methodology to ensure high internal validity by minimizing biases commonly associated with RCTs, including proper randomization and maintaining low attrition for both intervention and outcome assessments. Second, the research nurses monitored the exercises, ensuring appropriate compliance, intensity, and frequency of home-based exercises. Third, the personalized exercise program was designed for home implementation based on previous research and clinical knowledge. This program allowed for gradual increases in exercise load, with patients adjusting their exercise intensity based on their perceived exertion. With guidance materials, older patients could conduct the exercises at home, making this program feasible for implementation not only in specialized hospitals but also in clinics.

There are some limitations in the present study. First, due to the nature of the intervention, patients could not be blinded to the exercise intervention. Second, this was conducted as a single institutional study; therefore, validation using other cohorts is warranted. Third, we terminated the recruitment at 140 patients before reaching the initially planned 160 patients because the attrition rate of 5% was much lower than the estimated attrition rate of 20%, and the estimated sample size of 128 patients was achieved earlier. However, we believe this had little effect on the results.

Sarcopenia status was assessed at baseline to characterize the study population; however, changes in sarcopenia status were beyond the scope of the present study, which focused on changes in physical function assessed by the SPPB.

In conclusion, this RCT suggests that individualized exercise interventions for older patients with RA may improve physical function, muscle strength, and mental health-related QoL without exacerbating disease activity. Such exercise interventions may contribute to the comprehensive management of patients with RA.

## Supplementary Information

Supplementary Material 1. LSM change in SPPB scores at 8 and 16 weeks in patients with remission (DAS-CRP <2.8): subgroup analysis.

Supplementary Material 2. LSM change in RA disease activity.

Supplementary Material 3. LSM change in RA disease activity.

## Data Availability

The datasets supporting the conclusions of this article are included within the article and its additional files.
