# Supplementary material for: Effects of a personalized exercise program on physical function in older patients with rheumatoid arthritis at high risk of sarcopenia: results of a randomized controlled trial
Source: Arthritis Res Ther. 2026 Feb 6;28:66. doi: 10.1186/s13075-026-03751-8 (PMC12977742; doi:10.1186/s13075-026-03751-8)
Supplement: Supplementary file 1 — Supplementary Material 1. LSM change in SPPB scores at 8 and 16 weeks in patients with remission (DAS-CRP <2.8): subgroup analysis. [file 13075_2026_3751_MOESM1_ESM.docx]

Additional File 1. LSM changes in SPPB scores at 8 and 16 weeks in patients with remission (DAS28-CRP <2.6): subgroup analysis

| Variables | DAS | Week | Intervention (n=69) | | | Control (n=65) | | | Comparison between groups | |
| --- | --- | --- | --- | --- | --- | --- | --- | --- | --- | --- |
|  |  |  | N | Mean (SD) | LSM changes  (95% CI) | N | Mean (SD) | LSM changes  (95% CI) | Difference in LSM changes (95% CI) | p-value |
| SPPB | < 2.6 | 0 | 48 | 10.9 (1.4) |  | 37 | 10.8 (1.8) |  |  |  |
| total score |  | 8 | 45 | 11.3 (1.3) | 0.3 (0.0, 0.6) | 37 | 10.9 (1.6) | 0.0 (-0.3, 0.3) | 0.3 (-0.1, 0.8) | 0.136 |
|  |  | 16 | 47 | 11.4 (0.8) | 0.5 (0.2, 0.7) | 38 | 10.9 (1.5) | 0.2 (-0.1, 0.4) | 0.3 (0.0, 0.6) | 0.083 |
|  | ≥ 2.6 | 0 | 15 | 9.9 (2.4) |  | 17 | 10.7(1.6) |  |  |  |
|  |  | 8 | 15 | 10.0 (2.5) | 0.0 (-0.6, 0.6) | 15 | 10.7 (1.4) | 0.1 (-0.5, 0.7) | -0.2 (-0.1, 0.7) | 0.707 |
|  |  | 16 | 15 | 10.3 (2.8) | 0.2 (-0.5, 1.0) | 15 | 11.0 (1.1) | 0.2 (-0.5, 0.9) | 0.1 (-0.9, 1.1) | 0.871 |
| SPPB | < 2.6 | 0 | 48 | 3.7 (0.6) |  | 37 | 3.6 (0.6) |  |  |  |
| balance score |  | 8 | 46 | 3.7 (0.6) | 0.0 (-0.2, 0.2) | 38 | 3.6 (0.7) | -0.1 (-0.3, 0.2) | 0.1 (-0.2, 0.4) | 0.535 |
|  |  | 16 | 47 | 3.7 (0.6) | 0.1 (-0.1, 0.2) | 38 | 3.6 (0.6) | 0.0 (-0.2, 0.2) | 0.1 (-0.1, 0.3) | 0.426 |
|  | ≥ 2.6 | 0 | 15 | 3.4 (1.0) |  | 17 | 3.7 (0.6) |  |  |  |
|  |  | 8 | 15 | 3.4 (0.9) | -0.1 (-0.4, 0.2) | 15 | 3.7 (0.5) | 0.1 (-0.2, 0.4) | -0.2 (-0.7, 0.2) | 0.334 |
|  |  | 16 | 15 | 3.2 (1.2) | -0.3 (-0.7, 0.1) | 15 | 3.8 (0.4) | 0.1 (-0.3, 0.5) | -0.4 (-1.0, 0.2) | 0.194 |
| SPPB | < 2.6 | 0 | 48 | 3.8 (0.5) |  | 39 | 3.7 (0.6) |  |  |  |
| Gait speed score |  | 8 | 46 | 3.8 (0.2) | 0.1 (0.0, 0.2) | 38 | 3.8 (0.4) | 0.1 (0.0, 0.2) | 0.0 (-0.1, 0.1) | 0.904 |
|  |  | 16 | 47 | 3.9 (0.4) | 0.1 (0.0, 0.2) | 38 | 3.8 (0.5) | 0.1 (0.0, 0.3) | 0.0 (-0.1, 0.1) | 0.867 |
|  | ≥ 2.6 | 0 | 15 | 3.4 (0.9) |  | 17 | 3.8 (0.5) |  |  |  |
|  |  | 8 | 15 | 3.5 (0.8) | 0.1 (-0.1, 0.2) | 16 | 3.9 (0.3) | 0.1 (0.0, 0.3) | -0.1 (-0.3, 0.2) | 0.543 |
|  |  | 16 | 15 | 3.7 (0.7) | 0.2 (0.1, 0.4) | 17 | 3.9 (0.2) | 0.2 (0.0, 0.4) | 0.0 (-0.3, 0.3) | 0.954 |
| SPPB | < 2.6 | 0 | 48 | 3.4 (0.8) |  | 39 | 3.4 (0.9) |  |  |  |
| chair-stand test score |  | 8 | 46 | 3.7 (0.8) | 0.2 (0.0, 0.5) | 38 | 3.4 (1.0) | 0.0 (-0.3, 0.2) | 0.3 (-0.1, 0.6) | 0.112 |
|  |  | 16 | 47 | 3.7 (0.6) | 0.3 (0.1, 0.4) | 38 | 3.5 (0.9) | 0.1 (-0.1, 0.2) | 0.2 (0.0, 0.5) | 0.053 |
|  | ≥ 2.6 | 0 | 15 | 3.1 (1.4) |  | 17 | 3.2 (1.1) |  |  |  |
|  |  | 8 | 15 | 3.1 (1.3) | -0.1 (-0.5, 0.4) | 16 | 3.2 (1.2) | 0.0 (-0.4, 0.5) | -0.1 (0.7, 0.6) | 0.803 |
|  |  | 16 | 15 | 3.4 (1.2) | 0.3 (-0.1, 0.6) | 17 | 3.2 (1.1) | 0.0 (-0.3, 0.4) | 0.3 (-0.3, 0.8) | 0.315 |

DAS28-CRP, Disease Activity Score 28 joints; SPPB, Short Physical Performance Battery; LSM, least-squares mean; SD, standard deviation; CI, confidence interval.
