# Supplementary material for: Effects of a personalized exercise program on physical function in older patients with rheumatoid arthritis at high risk of sarcopenia: results of a randomized controlled trial
Source: Arthritis Res Ther. 2026 Feb 6;28:66. doi: 10.1186/s13075-026-03751-8 (PMC12977742; doi:10.1186/s13075-026-03751-8)
Supplement: Supplementary file 2 — Supplementary Material 2. LSM change in RA disease activity. [file 13075_2026_3751_MOESM2_ESM.docx]

Additional File 2. LSM changes in raw performance measures of SPPB components at 8 and 16 weeks

| Variables | Week | Intervention (n=69) | | | Control (n=65) | | | Comparison between groups | |
| --- | --- | --- | --- | --- | --- | --- | --- | --- | --- |
|  |  | N | Mean (SD) | LSM changes  (95% CI) | N | Mean (SD) | LSM changes  (95% CI) | Difference in LSM changes (95% CI) | p-value |
| SPPB | 0 | 68 | 9.8 (0.9) |  | 63 | 10.0 (0.0) |  |  |  |
| Semi-tandem, seconds | 8 | 67 | 10.0 (0.2) | 0.0 (-0.1, 0.2) | 62 | 9.9 (0.7) | 0.0 (-0.1, 0.1) | 0.1 (-0.1, 0.2) | 0.542 |
|  | 16 | 68 | 9.7 (1.3) | -0.2 (-0.4, 0.0) | 62 | 10.0 (0.0) | 0.1 (-0.2, 0.3) | -0.3 (-0.6, 0.1) | 0.098 |
| SPPB | 0 | 68 | 8.7 (2.6) |  | 64 | 8.4(2.8) |  |  |  |
| Tandem, seconds | 8 | 67 | 8.6 (2.7) | -0.1 (-0.7, 0.5) | 63 | 8.5 (2.8) | 0.0 (-0.6, 0.6) | -0.1 (-0.9, 0.8) | 0.838 |
|  | 16 | 68 | 8.6 (2.8) | 0.0 (-0.5, 0.5) | 62 | 8.7 (2.5) | 0.2 (-0.4, 0.8) | -0.2 (-1.0, 0.6) | 0.632 |
| SPPB | 0 | 68 | 1.1 (0.3) |  | 65 | 1.1 (0.3) |  |  |  |
| Gait speed, m/sec | 8 | 68 | 1.1 (0.2) | 0.0 (0.0, 0.0) | 64 | 1.1 (0.3) | 0.0 (-0.1, 0.0) | 0.0 (0.0, 0.1) | 0.565 |
|  | 16 | 68 | 1.1 (0.2) | 0.0 (0.0, 0.1) | 65 | 1.1 (0.3) | 0.0 (0.0, 0.0) | 0.0 (0.0, 0.1) | 0.215 |
| SPPB | 0 | 68 | 11.2 (4.0) |  | 65 | 3.7 (3.3) |  |  |  |
| Chair Stand, seconds | 8 | 66 | 9.8 (3.0) | -1.1 (-1.7, -0.5) | 62 | 3.7 (3.6) | 0.1 (-0.5, 0.7) | -1.2 (-2.0, -0.3) | 0.008 |
|  | 16 | 67 | 9.3 (2.9) | -1.6 (-2.1, -1.1) | 64 | 3.8 (3.7) | -0.1 (-0.6, 0.4) | -1.5 (-2.2, 0.8) | <.001 |

SPPB, Short Physical Performance Battery; Values represent raw performance measures, including balance times (semi-tandem stand and tandem stand, seconds), gait speed (m/s), and chair-stand test time (seconds). Side-by-side stand was not included in this table because all participants achieved the maximum performance at all time points (mean [SD] = 10.0 [0.0]), resulting in no variability.
