# Supplementary material for: Effects of a personalized exercise program on physical function in older patients with rheumatoid arthritis at high risk of sarcopenia: results of a randomized controlled trial
Source: Arthritis Res Ther. 2026 Feb 6;28:66. doi: 10.1186/s13075-026-03751-8 (PMC12977742; doi:10.1186/s13075-026-03751-8)
Supplement: Supplementary file 3 — Supplementary Material 3. LSM change in RA disease activity. [file 13075_2026_3751_MOESM3_ESM.docx]

Additional File 3. LSM changes from baseline in RA disease activity and functional status (DAS28, CDAI, SDAI, and HAQ) at 8 and 16 weeks

| Variables | Week | Intervention (n=69) | | | Control (n=65) | | | Comparison between groups | |
| --- | --- | --- | --- | --- | --- | --- | --- | --- | --- |
|  |  | N | Mean (SD) | LSM changes  (95% CI) | N | Mean (SD) | LSM changes  (95% CI) | Difference in LSM changes (95% CI), | p-value |
| DAS28-CRP | 0 | 63 | 2.1 (0.8) |  | 56 | 2.1 (0.7) |  |  |  |
|  | 8 | 59 | 3.2 (0.9) | 1.1 (0.9, 1.3) | 53 | 3.2 (0.9) | 1.0 (0.8, 1.2) | 0.1 (-0.2, 0.4) | 0.436 |
|  | 16 | 60 | 3.2 (0.8) | 1.0 (0.8, 1.2) | 56 | 3.0 (0.9) | 0.8 (0.6, 1.0) | 0.2 (-0.1, 0.5) | 0.156 |
| CDAI | 0 | 63 | 6.0 (7.1) |  | 57 | 5.5 (4.5) |  |  |  |
|  | 8 | 60 | 6.0 (7.1) | -0.2 (-1.2, 0.9) | 53 | 5.7 (6.0) | 0.0 (-1.1, 1.1) | -0.2 (-1.7, 1.3) | 0.794 |
|  | 16 | 59 | 5.4 (4.7) | -0.2 (-1.2, 0.7) | 56 | 4.9 (4.6) | -1.1 (-2.1, -0.1) | 0.9 (-0.5, 2.3) | 0.212 |
| SDAI | 0 | 63 | 6.2 (7.1) |  | 56 | 5.9 (4.7) |  |  |  |
|  | 8 | 59 | 6.2 (7.3) | -0.1 (-1.2, 1.0) | 53 | 6.1 (6.1) | 0.1 (-1.1, 1.3) | -0.2 (-1.8, 1.4) | 0.820 |
|  | 16 | 59 | 5.6 (4.8) | -0.2 (-1.2, 0.8) | 56 | 5.2 (4.8) | -1.2 (-2.2, -0.1) | 1.0 (-0.5, 2.4) | 0.195 |
| HAQ | 0 | 69 | 0.7 (0.5) |  | 65 | 0.7 (0.5) |  |  |  |
|  | 8 | 68 | 0.7 (0.6) | 0.0 (0.0, 0.1) | 64 | 0.7 (0.5) | 0.0 (-0.1, 0.0) | 0.1 (0.0, 0.1) | 0.044 |
|  | 16 | 68 | 0.7 (0.6) | 0.0 (0.0, 0.1) | 65 | 0.7 (0.5) | -0.1 (-0.1, 0.0) | 0.1 (0.0, 0.1) | 0.061 |

RA, rheumatoid arthritis; DAS28, disease activity score using 28 joints; CDAI, Clinical Disease Activity Index; SDAI, Simplified Disease Activity Index; HAQ, Health Assessment Questionnaire; LSM, least-squares mean; SD, standard deviation; CI, confidence interval.
